# Independent effects of bottom-up temporal expectancy and top-down spatial attention. An audiovisual study using rhythmic cueing

**DOI:** 10.3389/fnint.2014.00096

**Published:** 2015-01-06

**Authors:** Alexander Jones

**Affiliations:** Department of Psychology, Middlesex UniversityLondon, UK

**Keywords:** entrainment, crossmodal, endogenous, exogenous, attention, expectancy, hazard function

## Abstract

Selective attention to a spatial location has shown enhanced perception and facilitate behavior for events at attended locations. However, selection relies not only on where but also when an event occurs. Recently, interest has turned to how intrinsic neural oscillations in the brain entrain to rhythms in our environment, and, stimuli appearing in or out of sync with a rhythm have shown to modulate perception and performance. Temporal expectations created by rhythms and spatial attention are two processes which have independently shown to affect stimulus processing but it remains largely unknown how, and if, they interact. In four separate tasks, this study investigated the effects of voluntary spatial attention and bottom-up temporal expectations created by rhythms in both unimodal and crossmodal conditions. In each task the participant used an informative cue, either color or pitch, to direct their covert spatial attention to the left or right, and respond as quickly as possible to a target. The lateralized target (visual or auditory) was then presented at the attended or unattended side. Importantly, although not task relevant, the cue was a rhythm of either flashes or beeps. The target was presented in or out of sync (early or late) with the rhythmic cue. Results showed participants were faster responding to spatially attended compared to unattended targets in all tasks. Moreover, there was an effect of rhythmic cueing upon response times in both unimodal and crossmodal conditions. Responses were faster to targets presented in sync with the rhythm compared to when they appeared too early in both crossmodal tasks. That is, rhythmic stimuli in one modality influenced the temporal expectancy in the other modality, suggesting temporal expectancies created by rhythms are crossmodal. Interestingly, there was no interaction between top-down spatial attention and rhythmic cueing in any task suggesting these two processes largely influenced behavior independently.

## Introduction

Our sensory system is constantly exposed to vast amounts of information. To efficiently deal with this information and guide behavior we need to select, prioritize and predict certain events and stimuli over others. The collective term for this selective mechanism is known as attention. There are many forms of attention and one of the most extensively researched is how we focus our attention towards different locations in space. Spatial attention research is typically divided into endogenous attention, which is under voluntary control and exogenous attention which is bottom-up and stimulus driven. The most common method to explore the behavioral effects of endogenous and exogenous attention has been using the Posner cueing task (Posner, 1980). The participant’s task is to respond as quickly as possible to a target, usually presented peripherally to the left or right. In an endogenous version, the targets are preceded by a cue, usually centrally located, informing the most likely location of the target (70–80% likelihood). In an exogenous version, the cue, usually peripheral, does not give any indication of where the target may appear, however, the cue nevertheless typically elicits effects on target processing (Santangelo and Spence, 2008). Endogenous spatial attention has been studied extensively within and across modalities. Selective attention to a spatial location has shown to enhance perceptual processing (e.g., Mangun and Hillyard, 1990; Yeshurun and Carrasco, 1998) as well as facilitate response times (e.g., Posner et al., 1980) to visual stimuli at attended as compared to unattended locations (for a recent review see Carrasco, 2014).

Predictions about events in our environment rely not only on *where* something happens but also *when* an event occurs. Similar to spatial attention, focusing attention to a specific moment in time influences perception and biases our actions (Nobre, 2010). Temporal expectation can be generated in different ways and similar to voluntary spatial attention, instructive cues have been used to manipulate temporal expectations (e.g., Coull and Nobre, 1998; Naccache et al., 2002; Davranche et al., 2011; Zanto et al., 2011; Rohenkohl et al., 2014; see Nobre and Rohenkohl, 2014 for a recent review). Coull and Nobre (1998) used a symbolic cue to indicate when an upcoming target would likely appear, either 300 ms or 1500 ms after cue onset. In this detection task they found behavioral benefits when the cue appeared at a temporally anticipated compared to an unexpected time interval. Zanto et al. (2011) extended these findings showing similar benefits of voluntary orienting to targets at a particular point in time using event-related potentials (ERPs) as well as behavioral discrimination and Go-NoGo tasks. Moreover, Correa et al. (2005) showed high temporal expectancies increased perceptual sensitivity (d’) for detecting visual targets. Temporal cueing studies in the auditory modality have also shown effects of perceptual modulation by temporal cues. Several studies have observed a modulation of the early N1 component, suggested to originate from the primary auditory cortex, in response to temporally expected compared to unexpected tones (Lange and Röder, 2006; Lange et al., 2006; Lampar and Lange, 2011). There is thus mounting evidence showing that voluntary directing attention to a specific point in time influences both perception and modulates behavior.

Temporal expectancies can also be created by rhythms, something which commonly appears in our environment. For example the rhythm of breathing or our heartbeat, or the swaying of a tree, the sound and movement of walking or waves on a beach, the rhythmic structure of speech, or of course the rhythm in music. Rhythmic cueing has been used to investigate how external rhythms influences perception and performance. For example, Jones et al. (2002) presented participants with a standard tone which was followed by a sequence of tones presented in a rhythm. The participant’s task was to judge whether a target tone had the same pitch as the standard tone. They found that performance accuracy was better when the target tone was presented *in* compared to *out of* sync with the preceding rhythm. Auditory perceptual discrimination has consistently shown to be better when stimuli coincide with the rhythm and perceptual performance deteriorates if the stimuli is presented too early or too late in relation to the rhythm (Jones et al., 2006; see Jones, 2010 for a review). Similarly, response times have also been reported to be improved for stimuli occurring on the beat of a particular rhythm compared to an asynchronous rhythm using both auditory (e.g., Sanabria et al., 2011) or visual stimuli (Doherty et al., 2005; Rohenkohl et al., 2012; Cravo et al., 2013).

More recently the concept of rhythmic cueing has seen an increased research interest from a more neuroscientific viewpoint in that intrinsic brain operations are profoundly rhythmic (Raichle, 2010). Groups of neurons in the brain fluctuate rhythmically together and create oscillations with different frequencies which can be measures using electroencephalogram (EEG). These self-generated brain oscillations have shown to modulate responses and influence motoric, perceptual and cognitive processes (Buzsaki, 2006; Thut and Miniussi, 2009). It has for example been shown that the threshold of detecting visual stimuli fluctuates over time along with the phase of ongoing EEG activity (Busch et al., 2009). Importantly, the neural oscillation can also entrain to external rhythms aligning the firing pattern according to rhythms in our environment (Arnal and Giraud, 2012). In other words, neurons start to fire in synchrony with external rhythms. Moreover, entrainment to particular rhythms has been suggested to underlie selective attention (Lakatos et al., 2013; Calderone et al., 2014). For example Lakatos et al. (2008) presented monkeys with auditory and visual interleaved rhythms and found selectively attention to one stream amplified neural responses to events in that stream. Moreover, entrainment has been shown to increase with participant effort (Lakatos et al., 2013). This further indicates entrainment can also be modulated by higher level processes, such as attention. Similar to spatial attention, temporal expectancies can be bottom-up or top-down. However, it remains to be fully established to what extent rhythmic cueing and entrainment occurs unintentionally, in a purely bottom-up fashion. Recent evidence suggests temporal expectancies do still occur as a result of a rhythm even though the rhythm is detrimental to the task, suggesting automatic effects of rhythmic stimuli in the absence of top-down processes (Breska and Deouell, 2014).

Predicting where or when an event will occur has independently been shown to influence perception and drive behavior. However, space and time are not dimensions which occur in isolation in our environment, yet only a handful of studies have explored these two types expectation together. Doherty et al. (2005) manipulated both temporal and spatial expectancies by presenting participants with a ball which moved from left to right across a screen. Towards the right side of the screen there was a section which occluded the ball before reappearing. Doherty et al. found that response times were faster when the ball reappeared behind the occluding band in sync with the preceding rhythm. Similarly, response times were faster when the ball reappeared in the spatial location which was predicted by the balls trajectory across the screen. The individual effects were also additive showing faster response times when both temporal and spatial expectancies matched, an additive effect also demonstrated on the visual P1 component. Recently Rohenkohl et al. (2014) also investigated the synergy between spatial and temporal expectancies. In their task a symbolic visual arrow simultaneously indicated the likely location of a target as well as the likely time point when to expect the target. Unlike Doherty et al. they found an interaction between spatial and temporal effects. Temporal expectations improved visual perception, but only at spatially attended and not unattended locations. Importantly, in both studies (see also Tang et al., 2013) participants were asked to use both types of expectancies to increase performance. That is, both temporal and spatial expectancies were generated top-down, or, in Doherty et al. (2005) study using a rhythmic cue, a likely mix of stimulus and voluntary temporal attention. What remains less clear is how stimulus driven temporal expectancies, created by external rhythms, are affected by top-down spatial attention.

Crossmodal spatial attention effects have been extensively reported and shown to enhance perceptual processing and facilitate behavior (Vroomen and de Gelder, 2000; Spence and Driver, 2004). However, less is known how entrainment operates across modalities. In a study by Lakatos et al. (2007) it was observed that somatosensory inputs can reset the phase of the neural oscillations in primary auditory cortex of macaque monkeys, and in turn, auditory stimuli are enhanced or suppressed according to when in the oscillation they appear (see Kayser et al., 2008, for similar results in humans). This observation indicating that oscillations show crossmodal effects at a neural level. Moreover, recently Miller et al. (2013) also showed a crossmodal effect of entrainment whereby eye movements towards a visual target were faster if they occurred in sync with a preceding rhythm of tones.

The present study investigated how voluntary spatial attention affected automatic effects of rhythmic cueing using a simple detection task. Participants performed a typical Posner cueing task where an informative cue indicated to which side the target was most likely to appear. In addition, the cue consisted of four or five stimuli presented in a rhythm and the target was presented in or out of sync with this rhythm. Importantly this rhythm and the timing of the target was not task relevant. This novel paradigm allowed independent manipulation of top-down spatial attention and bottom-up temporal expectancies in order to investigate whether these represent dependent or independent mechanisms in driving behavior (as measured by response times). Furthermore, this study aimed to investigate whether rhythmic stimuli in one modality automatically influence the temporal expectancy in another modality. In separate tasks, participants were either presented with a visual cue and a visual target (VV), auditory cue and auditory target (AA) or in a crossmodal setting with a visual cue and an auditory targets (VA), or auditory cue and visual target (AV). Taken together, this study explored how endogenous spatial attention and stimulus-driven temporal expectancies, two processes which have independently shown to modulate perception and behavior, affected behavior in both a unimodal and crossmodal setting.

## Methods

### Participants

The study consisted 16 participants in each task, 64 in total (16 males; 13 right-, 3 left-handed, and 48 females; 43 right-, 5 left-handed). The participants were naive to the study and participated voluntarily or in return for course credits. The participant number was based on similar behavioral studies (e.g., Lawrence and Klein, 2013). Each participant only took part in one of the following four tasks: AA, AV, VA, VV. Due to excessive responses to catch trials and/or an inability to perform the task two participants were removed and replaced in the AV task, one from the VA, and two from the VV. The study was approved by the Middlesex University ethics committee and all participants provided written informed consent.

### Stimuli and materials

**Figure 1:**
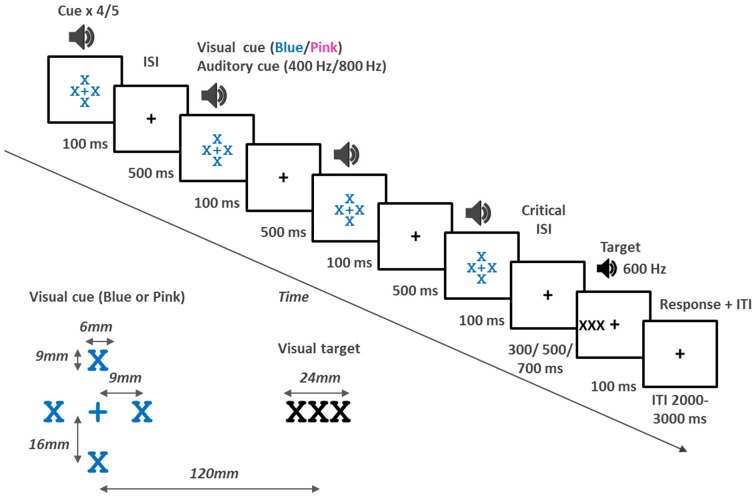
***Top*: Schematic view of events in a trial**. Each trial started with a cue which consisted of four or five interpolated stimuli, each 100 ms in duration, and presented with a 500 ms inter-stimulus interval (ISI) (SOA of 600 ms). In the VA and VV tasks the cue was either blue or pink Xs surrounding the fixation cross. The color of the cue indicated to which side to attend. In the AV and AA tasks the cue, which was either high or low frequency tones, indicated whether to attend to the left or right. The critical ISI was the interval between the last cue stimulus and the target. The target was 100 ms in duration. In the AV and VV tasks, the target was three X’s which appeared to the left or right of the fixation cross. In the VA and AA tasks the cue was a 600 Hz tone presented to either the left or right ear. The participant responded by pressing a key on the keyboard. After a response an inter trial interval of between 2000–3000 ms followed. A fixation cross was presented throughout in all tasks. ***Bottom left*:** Schematic representation of the visual cue and visual target as it appeared on screen.

The stimuli were presented and data collected using E-Prime v2 software (Psychology Software tools) run on a PC. Visual stimuli (fixation cross, visual cues and targets) were presented on a 17 inch monitor (1280 × 1024 pixels). A black fixation cross was presented in the middle of the screen. The visual cue consisted of an X above, below, to the left and right of the fixation cross creating the appearance of a larger cross in the center (see Figure 1 for details). Visual targets (three black Xs) were presented to the left or right side of the monitor. The font was Courier new. The participant was seated with their eyes approximately in line with the fixation cross and approximately 400–500 mm away from the screen. The visual angle for the target typically ranged between 18.15 and 14.7°. Auditory stimuli were presented via headphones (Audio 355, Plantronics). Auditory stimuli were 100 ms in duration with a 5 ms rise and fall time. The cue consisted of either low tones (400 Hz) or high frequency tones (800 Hz) and always presented in stereo. Targets were presented to only one ear and were 600 Hz. A keyboard was used to collect response times. The *down arrow* key was positioned in a straight line behind the fixation cross.

### Design and procedure

On each trial a rhythmic cue was presented. In the VA and VV task the color of the flashes (pink or blue) indicated whether the participant was to direct attention to the left or right. In the AV and AA tasks, the cue was either high or low tones, and this indicated which side attention was to be directed. A target then appeared at the attended (75%) or unattended side (25%) and the participant was to respond as quickly as possible by pressing the keyboard once a target appeared. In the AV and VV tasks, the target appeared to the left or right of the fixation cross and in the VA and AA tasks, the target was a tone presented to the left or right ear. The target appeared either in sync with the rhythm (the cue) or out of sync (early or late). The participant was not informed about this and it was not relevant to the task.

Each task consisted of five blocks with a total 260 trials (52 trials per block). Out of the 260 trials, 180 were attended (69%) and 60 unattended (23%), and 20 catch trials (8%). The weighting of targets, excluding catch trials was, 75/25 for attended/unattended targets. There was an even distribution of early, sync, and late trials. That is, for attended trials, there were 60 early, 60 sync, and 60 late trials per participant, and 20 unattended trials for each of the early, sync and late conditions. For half of the trials the cue consisted of four stimuli, and for half of the trials the rhythmic cue included five stimuli. Prior to the experiment the participant ran a practice block.

The participant was seated in an experimental booth in front of a PC monitor. In the tasks including auditory stimuli (all but the VV task) the participant wore headphones. Each trial started with the presentation of the rhythmic cue which consisted of four or five stimuli presented every 600 ms (see Figure 1 for events in a trial). More specifically, the first of the rhythmic stimuli (the cue) was presented for 100 ms followed by an inter-stimulus interval (ISI) of 500 ms. The cue stimuli were presented four of five times creating a rhythm of 100 ms stimulus every 600 ms. After the last of the interpolated stimuli a target was presented. The critical ISI, preceding the target was 300 ms (early), 500 ms (sync), or 700 ms (late). The target was then presented for 100 ms. Participants responded with their dominant hand by pressing the *down arrow* key on the keyboard. If no response was recorded the trial terminated after 2000 ms. There was a random inter-trial interval of 2000–3000 ms. A centrally located fixation cross was presented throughout and participants were explicitly instructed to keep their gaze on this fixation cross at all times.

In the tasks with an auditory cue (AA and AV), for half the participants high frequency tones indicated to attend to the left and low tones indicated attend to the right. This allocation was reversed for the other half. Similarly, for half the participants who performed a task with a visual cue (VA and VV), a blue Xs indicated attend to the left and pink Xs attend to the right, and the reverse for the other half of participants.

The RT data was Log_10_-transformed and submitted to a mix design ANOVA with the factors Task (AA, AV, VA, VV), Spatial attention (attended, unattended), Temporal expectancy (early, sync, late), and Rhythm count (four stimuli, five stimuli). Following the overall analysis, each task was analyzed separately.

## Results

### Summary

The results showed that participants responded faster to attended compared to unattended trials in all four tasks. There was also an effect of temporal expectancy in all tasks but the unimodal auditory task (AA). In the two cross-modal tasks (AV and VA) the targets were faster when in sync and late targets compared to early targets. In the visual task the late targets were faster than both in sync and early targets. Although clear effects of spatial attention and temporal expectancy were observed there was no evidence of an interaction between these two factors in any task. Thus suggesting temporal expectancy and spatial attention affected response times independently.

#### Overall analysis including task

A mixed design ANOVA, including Task as a factor, showed a main effect of Spatial attention (*F*_(1,60)_ = 36.32, *p* < 0.001, $\eta_{p}^{2}$ = 0.38) with faster RTs for attended (325.2 ms) compared to unattended trials (367.6 ms). There was a main effect of Temporal expectancy (*F*_(2,120)_ = 27.27, *p* < 0.001, $\eta_{p}^{2}$ = 0.31) and follow up pairwise-comparisons (Bonferroni corrected) showed that sync (342.8 ms) and late targets (339.7 ms) were significantly faster than early targets (356.6 ms) (both *p*’s < 0.001). There was no difference between sync and late targets (*p* = 0.18). There was a main effect of Rhythm count (*F*_(1,60)_ = 42.32, *p* < 0.001, $\eta_{p}^{2}$ = 0.41) with targets preceded by four stimuli in the rhythm (352.8 ms) were slower compared to if the rhythmic cue contained five stimuli (340.0 ms). There was a main effect of Task (*F*_(3,60)_ = 6.24, *p* = 0.001, $\eta_{p}^{2}$ = 0.24) and Bonferroni *post hoc* test showed that the AV task was significantly faster (297.8 ms) compared to AA (380.5 ms) and VV task (369.1 ms) (*p* = 0.002 and *p* = 0. 005 respectively).

There was a Temporal expectancy*Task interaction (*F*_(6,120)_ = 3.30, *p* = 0.005, $\eta_{p}^{2}$ = 0.14) and planned analysis for each task is presented below. There was no Task*Spatial attention interaction (*p* = 0.59, $\eta_{p}^{2}$ = 0.04). Important to note is there was no Spatial attention*Temporal expectancy interaction (*p* = 0.37, $\eta_{p}^{2}$ = 0.02). There was a Rhythm count*Temporal expectancy interaction (*F*_(2,120)_ = 10.27, *p* < 0.001, $\eta_{p}^{2}$ = 0.15) suggesting the effect of Temporal expectancy was different according to the number of stimuli in the cue. This interaction will also be explored in the analysis of each task. No other main effects or interaction were significant.

### Visual cue—visual target (VV)

Overall participants missed less than 1% of targets and responded to 1.9% of catch trials in the VV task.

**Figure 2:**
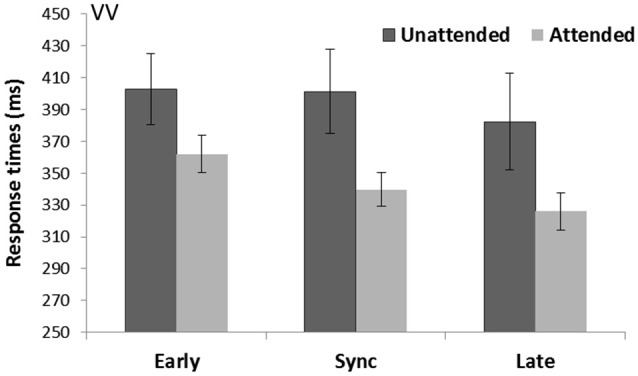
**Unimodal visual cue and visual targets task (VV)**. Mean response times (with standard error bars) for visual targets presented at spatially attended (light gray) and unattended side (dark gray), separately for early, sync and late conditions in relation to the visual rhythm (the cue). Response times were significantly faster for attended over unattended targets. There was a main effect of temporal expectancy where late targets were faster than both early and in sync targets, and in sync targets were also faster than early targets.

There was a significant effect of Spatial attention (*F*_(1,15)_ = 5.74, *p* = 0.03, $\eta_{p}^{2}$ = 0.28) with attended trials being faster (342.6 ms) than unattended trials (395.5 ms). The purely visual task showed a main effect of Temporal expectancy (*F*_(2,30)_ = 17.60, *p* < 0.001, $\eta_{p}^{2}$ = 0.54). Pairwise comparisons (Bonferroni corrected) demonstrated late targets (339.7 ms) to be faster (*p* = 0.002) than in sync targets (370.6 ms), and late targets were faster (*p* = 0.001) than early targets (382.4 ms), and in sync targets were also faster compared to early targets (*p* = 0.049; see Figure 2). There was a main effect of Rhythm count (*F*_(1,15)_ = 24.03, *p* < 0.001, $\eta_{p}^{2}$ = 0.62) with on average faster RTs for visual targets preceded by five stimuli (356.9 ms) compared to four stimuli (381.2 ms). There was no Spatial attention*Temporal expectancy interaction (*p* = 0.18, $\eta_{p}^{2}$ = 0.11).

### Auditory cue—auditory target (AA)

Overall participants missed 1.4% of targets and responded to 4.4% of catch trials in the AA task.

**Figure 3:**
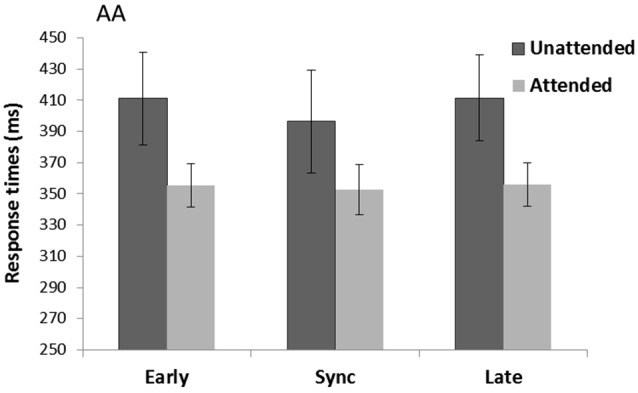
**Unimodal auditory cue and auditory targets task (AA)**. Mean response times (with standard error bars) for auditory targets presented at spatially attended (light gray) and unattended side (dark gray), separately for early, sync and late conditions in relation to the auditory rhythm (the cue). Response times were significantly faster for attended over unattended targets.

There was a significant main effect of Spatial attention (*F*_(1,15)_ = 8.99, *p* = 0.009, $\eta_{p}^{2}$ = 0.38) with faster RTs for attended (354.7 ms) compared to unattended trials (406.3 ms) (see Figure 3). There was also a main effect of Rhythm count (*F*_(1,15)_ = 8.15, *p* = 0.012, $\eta_{p}^{2}$ = 0.35) with overall faster RTs for when the cue consisted of five (376.7 ms) compared to four tones (384.3 ms). There was no main effect of Temporal expectancy (*p* = 0.59, $\eta_{p}^{2}$ = 0.03) or Spatial attention*Temporal expectancy interaction (*p* = 0.36, $\eta_{p}^{2}$ = 0.07).

### Auditory cue—visual target (AV)

Participants missed 2% of targets and responded to 4.4% of catch trials in the AV task.

**Figure 4:**
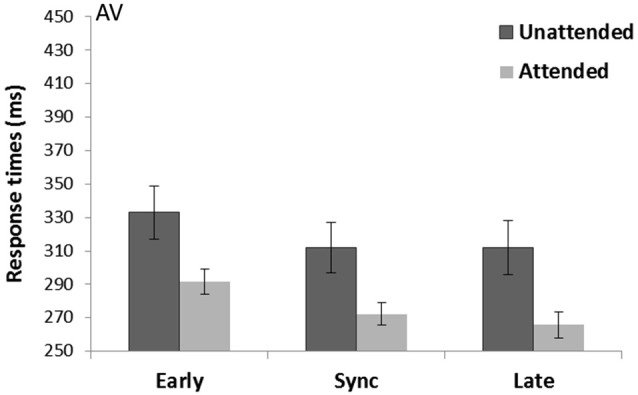
**Crossmodal auditory cue and visual targets (AV) task**. Mean response times (with standard error bars) for targets presented at spatially attended (light gray) and unattended side (dark gray), separately for early, sync and late conditions in relation to the auditory rhythm (the cue). Response times were significantly faster for attended over unattended visual targets. There was a main effect of Temporal expectancy where in sync and late targets were faster than early targets.

A main effect of Spatial attention (*F*_(1,15)_ = 26.07, *p* < 0.001, $\eta_{p}^{2}$ = 0.64) showed attended trials were faster (276.6 s) compared to unattended trials (319.0 ms). There was also a main effect of Temporal expectancy (*F*_(2,30)_ = 14.38, *p* < 0.001, $\eta_{p}^{2}$ = 0.49). Pairwise-comparisons (Bonferroni corrected) showed both sync (292.2 ms) and late targets (288.9 ms) were significantly faster than early targets (312.4 ms; *p* < 0.001 and *p* = 0.001 respectively; see Figure 4). There was no Spatial attention*Temporal expectancy interaction (*p* = 0.27, $\eta_{p}^{2}$ = 0.08).

There was a Rhythm count*Temporal expectancy interaction (*F*_(2,30)_ = 10.74, *p* = 0.002, $\eta_{p}^{2}$ = 0.42). Follow-up analysis of trials with a rhythm of four tones preceding the visual target showed an effect of Temporal expectancy (*F*_(1,15)_ = 20.56, *p* < 0.001, $\eta_{p}^{2}$ = 0.58). Pairwise-comparisons (Bonferroni corrected) showed a difference between early (327.9 ms) and in sync (296.4 ms) and early and late targets (288.8 ms) (both *p*’s < 0.001). When there were five tones in the rhythm, no effect of target Temporal expectancy was present (*p* = 0.14).

### Visual cue—auditory target (VA)

Overall participants missed less than 1% of targets and responded to 1.6% of catch trials in the VA task.

**Figure 5:**
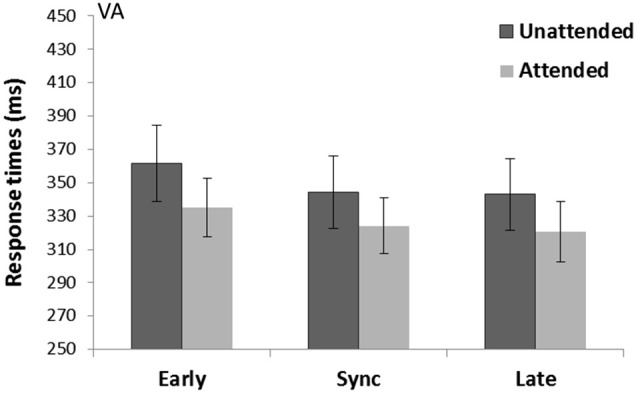
**Crossmodal visual cue and auditory targets task (VA)**. Mean response times (with standard error bars) for tones presented at spatially attended (light gray) and unattended side (dark gray), separately for early, sync and late conditions in relation to the visually presented rhythm (the cue). Response times were significantly faster for spatially attended over unattended targets and a main effect of Temporal expectancy showed in sync and late targets were faster than early targets.

A main effect of Spatial attention (*F*_(1,15)_ = 6.83, *p* = 0.02, $\eta_{p}^{2}$ = 0.31) revealed attended trials were faster (326.6 ms) compared to unattended trials (349.6 ms) (see Figure 5). There was also a main effect of Temporal expectancy (*F*_(2,30)_ = 9.75, *p* = 0.001, $\eta_{p}^{2}$ = 0.39) and pairwise-comparisons (Bonferroni corrected) showed in sync (370.6 ms) and late targets (354.3 ms) were faster than early target (382.4 ms) (*p* = 0.02 and *p* < 0.001 respectively). There was no Spatial attention*Temporal expectancy interaction (*p* = 0.72, $\eta_{p}^{2}$ = 0.02).

## Discussion

In four separate tasks, this study investigated the effects of voluntary spatial attention and bottom-up temporal expectancy in both unimodal and crossmodal conditions. In all tasks response times to targets were faster when they appeared at the attended compared to unattended location. This indicated that participants followed instructions and the results replicated what has previously been observed in unimodal visual (e.g., Posner, 1980; Wright and Ward, 1994) and auditory spatial attention tasks (Spence and Driver, 1994), as well as audiovisual crossmodal tasks (Spence and Driver, 1996; or Spence, 2010 for a review). The present study also demonstrated a main effect of temporal expectancy in both crossmodal tasks indicating that rhythmic stimuli in one modality automatically influenced the temporal expectancy in the other modality. An effect of rhythmic cueing was also observed in the unimodal visual task but not unimodal auditory task. Interestingly, there was no observed interaction between top-down spatial attention and temporal expectancy effects in any task suggesting temporal and spatial processing independently affected target response times.

That selectively attending to a spatial location enhances perceptual processing and facilitates behavior at the attended locations has been well documented (for a recent review see Carrasco, 2014). Although relatively less researched, voluntary temporal expectation has also been shown to influence perception and drive behavior (Nobre et al., 2011). Moreover, animal studies have demonstrated that temporal expectations can modulate neural processing in early sensory areas such as primary visual cortex (Lima et al., 2011) and primary auditory cortex (Jaramillo and Zador, 2011). As with spatial selective attention, temporal predictability can be divided into voluntary and stimulus-driven processes. Rhythmic cueing has been used to create temporal predictability, and the rhythm can be used to induce both voluntary or stimulus driven effects, or a combination of both, particularly depending on the instructions. In the present study the rhythmic structure of the cue was not task relevant, in order to investigate whether or not stimulus driven expectancies affected target processing. In other words, whether rhythmic cueing automatically influenced response times even when this temporal characteristic was not relevant or beneficial to the task.

In the unimodal visual task and the two crossmodal conditions the preceding rhythm influenced target detection times. In the visual task, responses to targets in sync with the rhythm were faster compared to early targets, and late targets were also faster compared to when the target was in sync with the rhythm. Similar effects of visual rhythmic cueing have been observed in a study by Rohenkohl et al. (2011) where participants attended to either the color or speed (rhythm) of a moving disc across the screen, to predict an upcoming target. They found both types of cue speeded up response times to targets. However, they found rhythmic cues facilitated response times, compared to an arrhythmic condition, regardless if the participant was instructed to use the rhythmic information or not, whilst the symbolic color cue was only effective when participants explicitly used this information. However, when using rhythmic stimuli to induce temporal expectancies, it may still be difficult to tear apart the contribution of purely bottom-up effects caused by the rhythm, and any top-down influence such as directing attention to a specific point in time. In other words, are temporal expectancies created passively and purely unintentionally when we are exposed to rhythmic patterns? In some studies using a rhythmic cue, the target was always in sync with the rhythm in the rhythmic condition, as compared to a non-rhythmic condition (e.g., Doherty et al., 2005; Rohenkohl et al., 2012) and in others, the target was more likely to occur in sync compared to out of sync with a rhythm (e.g., Praamstra et al., 2006). In other words, crating conditions where it would be advantageous to use the rhythm to anticipate targets even though not explicitly instructed to do so. Breska and Deouell (2014) specifically investigated whether a rhythm automatically creates temporal expectancies. They included a condition where it was detrimental to use the rhythm to perform the task but still found that the rhythm affected target detection, concluding rhythms automatically exerts an effect on target processing. In the present study the rhythm was not task relevant, but, participants were not explicitly discouraged from using the rhythm. However, the probability of the target appearing at early, sync or late intervals was equally likely and therefore any strategy of expecting the target at a particular time point would not be advantageous. This together with participants concurrently performing another task, directing spatial attention, suggests the rhythmic cueing effects were mainly bottom-up and not involving higher level of processing. However, future studies may wish to specifically address the automaticity of rhythmic cueing and how systematically varying the automaticity is affected by top-down spatial attention. In any case, it can be concluded from the present study that rhythmic cueing effects were observed even though participants performed a concurrent spatial attention task.

In rhythmic cueing studies the perception of targets is typically best when the stimulus coincides with the rhythm and perceptual benefits decrease if the stimulus is presented too early or too late (e.g., Jones et al., 2002; Mathewson et al., 2010). In the current study the detrimental effect of asynchronous stimuli was only present for early, but not for late stimuli. The target was equally likely to appear at early, sync or late intervals meaning there was no strategic benefit in expecting the target at any particular time. However, the pattern of results can in part be explained by the hazard or foreperiod effect. The “hazard function” is an effect whereby an event is more likely to occur at a specific point in time if it has not yet occurred (Luce, 1986). In other words, if the stimulus has not appeared at the early time point it is then more likely to occur at the sync and subsequently the late time interval. This in turn can increase the anticipation and enhance motor readiness. Several steps were taken to account for and to minimize this potential bias. First, catch trials were used whereby no target was presented. This introduces the possibility that if a target has not occurred in sync with the rhythm, it will not necessarily occur at the late time interval. Moreover, to further reduce the hazard function effects and to increase temporal uncertainty of when the target may occur, the cue randomly consisted of either four or five stimuli. Finally, the participants were not informed about the temporal manipulation of the study. Nevertheless, the hazard function fits well with the pattern observed in the purely visual task (VV) whereby response times decreased orderly from early, sync and then late conditions. The expected pattern of results in terms of a model of entrainment would be that in sync targets would be faster than both early and late targets. In the two cross modal tasks there was no difference between late and in sync targets which may suggest both hazard function and entrainment effects influenced RTs. That is, in the early condition both entrainment and hazard effects predict slower RTs compared to the in sync condition. However, when the target is late, the hazard function predicts faster RTs compared to in sync targets, whilst an entrainment model would predict slower RTs. It is therefore possible that both entrainment and hazard function effects were present in this study. Future research may wish to use target discrimination tasks or detection of targets at perceptual threshold to further isolate entrainment effects from hazard functions.

Both crossmodal tasks showed similar effects of rhythmic cueing with a facilitation of response times for targets coinciding with the rhythm compared to when they appeared early. Importantly, this shows that stimulus driven rhythmic cueing is not limited to within a specific modality but effects can span across modalities. This is in line with a recent study by Miller et al. (2013) who found saccades to a visual target were faster when the target was preceded by a synchronous compared to an asynchronous auditory rhythm (see also Bolger et al., 2013 for similar results). The present study extends their findings by showing that audiovisual effects of rhythmic cueing are also found when the modalities are reversed, that is, a visual rhythm entrains auditory targets. This may suggest for a common mechanisms of temporal expectancy created by rhythms which is not modality specific. In line with this, Besle et al. (2011) observed large scaled entrainment of brain areas using intracranial electrocortical recordings in patients with epilepsy. They specifically found that the entrainment of visual stimuli was not confined to the primary visual areas but was observed over a larger brain area. That is, they observed effects in line with a centralized rather than purely modality specific entrainment mechanism.

The one spurious result in the present study was the lack of an temporal expectancy effect in the unimodal auditory task. Auditory entrainment of rhythms has shown to affect target discrimination of tones (e.g., Jones et al., 2002), as well as response times (Sanabria et al., 2011). In contrast, finding an effect of spatial attention in an auditory detection task, as was observed here, has proven more difficult with many studies reporting a null result (e.g., Posner, 1978; Scharf et al., 1987; Buchtel and Butter, 1988; Hugdahl and Nordby, 1994; although see Spence and Driver, 1994 for a positive effect of auditory spatial attention). Whether the introduction of an auditory spatial task diminished any auditory temporal effects remains unclear, however it seems unlikely as the auditory rhythm in the crossmodal task led to temporal expectancy effects of visual stimuli.

The study aimed to investigate how two processes which have independently shown to influence perception and modulate behavior interacted. The results showed that in no task was there an interaction between spatial attention and rhythmic cueing (*p* = 0.37, $\eta_{p}^{2}$ = 0.02). In other words, any effects of rhythmic cueing were similar regardless if the target appeared at the spatially attended or unattended location. Whilst the results here show both unimodal and crossmodal effects of spatial attention, and unimodal and crossmodal effects of rhythmic cueing, spatial attention and temporal expectancy themselves did not interact, neither at a unimodal nor crossmodal level. This suggests temporal and spatial processes can operate independently in driving behavior, at least as measured with response times. Doherty et al. (2005) found similar independent effects of spatial attention and rhythmic cueing on response times, even though their participants were instructed to use the rhythmic structure to predict an upcoming target and thus introducing a voluntary aspect of rhythmic cueing. In contrast, Rohenkohl et al. (2014) recently showed temporal expectation improved perception when the target appeared at a spatially attended location. However, at unattended locations, temporal expectancy did not affect target processing. Rohenkohl et al. did not use rhythmic cueing but the temporal expectation was top-down. Moreover, they investigated perceptual sensitivity rather than response times which may also account for differences. Cravo et al. (2013) measured response times and perceptual accuracy and found both measured to be improved in a rhythmic compared to arrhythmic condition, but, the two measures showed independent effects. There is thus evidence to suggest perceptual modulation following rhythmic stimuli may be different to response time effects. Future research may wish to explore whether perceptual sensitivity effects of automatic entrainment are also independent from spatial attention effects in unimodal and crossmodal conditions. Moreover, the current study used a target detection task which was relatively easy. Within spatial attention research, endogenous and exogenous effects are typically independent when task demands are low. However, when the attentional and cognitive load increases, the two processes have shown to interact when competing for shared resources (Berger et al., 2005). It is conceivable that top-down spatial attention and bottom-up temporal expectancy effects show a similar pattern. In other words, future research could increase the difficulty of the task to investigate whether endogenous spatial attention and stimulus-driven temporal expectancies are independent even when demands on attentional resources are high.

The automatic effect of presenting rhythmic stimuli demonstrated in the present study is partly in line with research on neural oscillations which have seen a recent increase in popularity in the last decade. Evidence is mounting that, not only does our brain self-generate rhythmic oscillations which drives perception and action (e.g., Buzsaki, 2006; Thut and Miniussi, 2009), but these neural oscillations can also be re-set and driven by rhythms and events in our environment (Lakatos et al., 2008; Arnal and Giraud, 2012). Investigating the function and underlying mechanisms of entrainment will not only further our understanding of what drives our behavior and influences our perception, but recent findings have suggested that certain psychiatric and developmental disorders show abnormal neural oscillation patterns (see Calderone et al., 2014 for a recent review).

## Conflict of interest statement

The author declares that the research was conducted in the absence of any commercial or financial relationships that could be construed as a potential conflict of interest.
